# Non-Conventional Allogeneic Anti-BCMA Chimeric Antigen Receptor-Based Immune Cell Therapies for Multiple Myeloma Treatment

**DOI:** 10.3390/cancers15030567

**Published:** 2023-01-17

**Authors:** Zhicheng Du, Sumin Zhu, Xi Zhang, Zhiyuan Gong, Shu Wang

**Affiliations:** Department of Biological Sciences, National University of Singapore, Singapore 117543, Singapore

**Keywords:** non-conventional, off-the-shelf, immune cells, anti-BCMA CAR, multiple myeloma, clinical regulations

## Abstract

**Simple Summary:**

Multiple Myeloma (MM) is the second most common hematological malignancy in the world. The current two US Food and Drug Administration (FDA)-approved anti-BCMA chimeric antigen receptor (CAR)-T cells therapies for MM treatment rely on patients’ own peripheral blood T cells and can only be used for those patients. This type of personalized treatment has several limitations, including high costs, long manufacturing times, and possible manufacturing failure. To solve these problems, in this review, we introduced the possibility of applying other immune cells to equip anti-BCMA CARs to treat MM, which could potentially achieve the goal of “off-the-shelf”. Although many of these therapies are still in early development, some of them have already entered the clinical trial stage and shown promising results in terms of safety and patient survival.

**Abstract:**

MM, characterized by the progressive accumulation of clonal plasma cells in bone marrow, remains a severe medical problem globally. Currently, almost all MM patients who have received standard treatments will eventually relapse. Autologous anti-BCMA CAR-T cells are one of the FDA-approved immunotherapy cell-based products for treating adults with relapsed or refractory (r/r) multiple myeloma. However, this type of CAR-T cell product has several limitations, including high costs, long manufacturing times, and possible manufacturing failure, which significantly hinder its wider application for more patients. In this review, we summarized the current development stage of applying other types of immune cells to bring the anti-BCMA CAR-T therapy from autologous to allogeneic. In general, anti-BCMA CAR gene-edited αβ T cells and CAR-Natural Killer (NK) cells are at the forefront, with multiple clinical trials ongoing, while CAR-γδ T cells and CAR-invariant Natural Killer T (iNKT) cells are still in pre-clinical studies. Other immune cells such as macrophages, B cells, and dendritic cells have been mainly developed to target other antigens and have the potential to be used to target BCMA. Nevertheless, additional regulatory requirements might need to be taken into account in developing these non-conventional allogenic anti-BCMA CAR-based cell products.

## 1. Introduction

MM is the second most common hematological malignancy and is characterized by the progressive accumulation of clonal plasma cells in bone marrow [1,2]. In 2016, there were 138,509 cases of MM globally, a rise of 126% compared to figures from 1990 [3]. In contrast, the average mortality rate has decreased significantly, from 4.0/100,000 in 1994 to 3.2/100,000 in 2017, likely due to the advancement of the therapeutical armamentarium and increased survival [4]. In the US, 34,470 people are expected to be diagnosed with MM, and 12,640 deaths are expected to be confirmed for the previous year 2022 [5]. In China, these numbers were estimated to be 22,450 and 17,360 in 2022, respectively [6]. Nevertheless, MM is not a very common malignancy and only accounts for approximately 1% of all types of cancers, which indicates that the absolute number of affected patients of MM might not be a priority from the perspective of public health [7]. Indeed, the current main challenges of managing MM globally focus on three other aspects. Firstly, similar to many other cancers, the cost of treating MM remains a medical burden for most patients. According to a recent study conducted in the US, for older adults (mean = 75.8 years old) who have been diagnosed with MM, the incremental lifetime MM cost is $184,495, with the majority going to inpatient and outpatient expenditures and prescribed drugs [8]. Secondly, although therapies for MM have developed rapidly in recent years, not all patients are treated properly or can access those treatments. The possible reasons for this are complicated, including low socioeconomic development and delays in the inclusion of new therapies in national formularies by the government [3,9]. The last issue for treating MM is the problem of relapse and refractory (r/r). The current standard treatment for MM is “triplet regimens-based” [10]. In China, according to the latest version of the Chinese Society of Hematology’s guideline for the diagnosis and management of multiple myeloma, the combination of one proteasome inhibitor (PI, such as Bortezomib and Carfilzomib), one immunomodulatory drug (IMiD, such as Lenalidomide and thalidomide), and dexamethasone is recommended for newly diagnosed MM patients [11]. In the US, the treatment strategy is comparably more specific. Referring to the guideline from the National Comprehensive Cancer Network (NCCN), a combined therapy with Bortezomib, Lenalidomide, and dexamethasone was set as the preferred regimen for both autologous stem cell transplantation (ASCT)-eligible and -non-eligible MM patients [12]. However, almost all patients, including those who achieve complete remission (CR) after the initial treatments, will eventually relapse [10,13]. Compared to newly diagnosed MM, r/r MM is usually less responsive and easy to progress either during or shortly after treatment [14]. For patients in early relapses, different combinations of triplet regimens or monoclonal antibodies (mAbs) could be applied [11,12]. For example, Daratumumab, a type of mAbs targeting CD38, received approval from the US FDA for treating MM in 2015 [15]. For patients with late relapse, CAR-T therapies are usually recommended [11,12]. Until September 2022, there were already two anti-B cell maturation antigen (BCMA) CAR-T cell therapies that had been approved by the US FDA to treat adults with r/r multiple myeloma after four or more prior lines of therapy [16]. 

BCMA, also known as tumour necrosis factor receptor (TNFR) superfamily member 17, is a transmembrane glycoprotein mainly involved in the maturation, survival, and differentiation of B cells into plasma cells with the coordination of two other TNFR superfamily members [17]. It is known to be one of the most promising antigens for developing CAR-T therapy for MM treatment, given its universal overexpression in malignant plasma cells and restricted expression in B-cell lineage cells [18,19]. 

CAR is an artificial receptor designed to direct immune cells (e.g., T cells and NK cells) to cancer cells, stimulating the immune response to kill those target cells [20]. A CAR molecule typically comprises four parts: an antigen recognition domain, a hinge region, a transmembrane domain, and an intracellular signaling domain [21]. The antigen recognition domain is generally designed to recognize and bind to the tumour-associated or specific surface antigens on cancer cells [22]. It is usually adapted from mAb’s heavy and light chains, named single chain variable fragments (scFvs). However, in some cases, it can also be directly derived from native immune cell receptors such as the NKG2D [22,23]. The hinge region and transmembrane domain (H/T) are comparably more conserved and are usually obtained from a portion of widely expressed receptors on T cells, such as CD8 and CD28. In general, H/T provides the CAR with spatial flexibility and anchors the construct on the cell surface [24]. The intracellular signaling domain triggers intracellular cascade signaling and leads to subsequent anti-tumour effects of immune cells [24]. The first-generation CAR only contains one CD3-ζ domain derived from the cytoplasmic tail of the T cell’s receptor (TCR) in the intracellular signaling domain. For the second-generation CAR, a co-stimulatory molecule is incorporated to further sustain the immune cells’ responses and proliferation in vivo, including the most well-known CD28 and 4-1BB’s endodomains [25,26]. Although many other modifications have been initiated to further enhance CAR’s functions, such as introducing more than one co-stimulatory molecule or other cytokine inducers, the second-generation CAR construct with only one co-stimulatory domain is still considered today’s breakthrough in CAR immunotherapy for cancer. This is mostly because more modifications do not confer consistent improvements for CAR-equipped immune cells and instead may lead to cell exhaustion and terminal differentiation [27].

The current two FDA-approved anti-BCMA CAR-T cells are both second-generation autologous CAR-T cells. ABECMA (idecabtagene vicleucel) is the 1st cell-based therapy for treating MM. An open-label, single-arm, multicenter phase II clinical trial-KarMMa showed 72% of the overall response rate (ORR) and 11 months of median duration of response (DoR) for r/r MM patients who had received this treatment [28]. Soon after, CARVYKTI (ciltacabtagene autoleucel) also received approval based on the results of an encouraging phase I/II clinical study (CARTITUDE-1), with the ORR at 97.9% and a median DoR of 21.8 months, respectively [29]. Autologous CAR-T cell therapies usually possess a comparably good safety profile, with very limited graft versus host diseases (GvHD) and hyperreactive self-immune responses observed [28,29,30]. However, there are also a few limitations. Firstly, the cost of these cell products is high; this is mainly attributed to their sophisticated and bespoke manufacturing process [31]. The prices for one single infusion of the two anti-BCMA CAR-T cells products mentioned above are USD 441,743 and 489,654.5, which are not affordable for most families worldwide [32]. Secondly, the manufacturing of autologous CAR-T cells is also time-consuming. According to statistics from the FDA, an estimation of 4 to 5 weeks is typically required for the two anti-BCMA CAR-T cells to be produced [28,29]. This is a rather lengthy process, especially for r/r MM patients. As shown by the data from clinical trials, 4 out of 135 patients for ABECMA and 11 out of 113 patients for CARVYKTI failed to receive treatment due to death, adverse events, or disease progression [28,29]. The last major issue is the possible manufacturing failure of CAR-T cells. Autologous T cells must be produced from patients who have already experienced several rounds of other types of standard treatments. Hematological adverse drug reactions, especially leukopenia, are the most commonly reported problems for patients taking PI and IMiD drugs, which can significantly affect the quality of the collected cells [33,34]. Referring to the clinical studies’ results, 1.5% (2/135) and 18% (17/97) manufacturing failures were reported for ABECMA and CARVYKTI, respectively [28,29]. Besides the quality problems regarding donor blood, other factors such as the contamination of pathogens or myeloma cells could also give rise to manufacturing risks and eventually lead to manufacturing failure [35].

**Figure 1:**
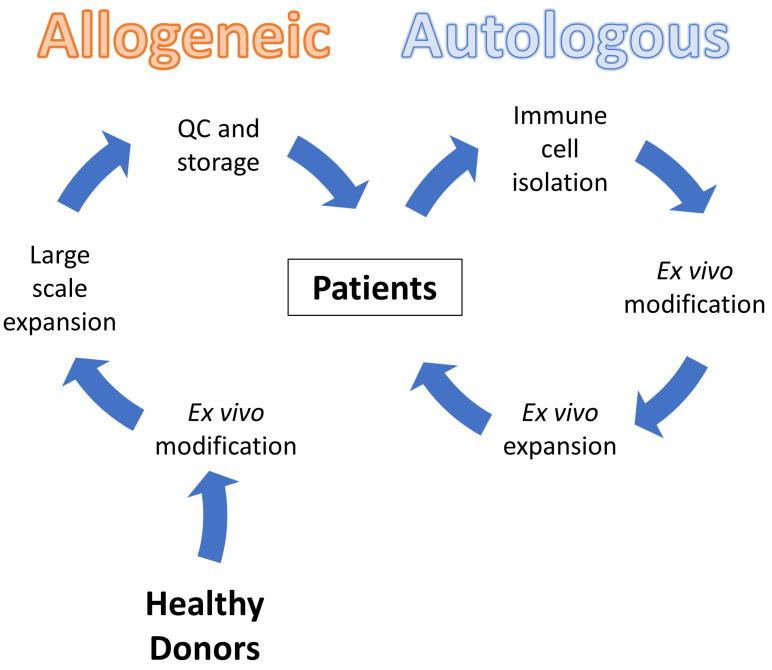
Allogeneic and autologous adoptive transfer of CAR-based immune cells.

To address the above-listed limitations, recently, scientists have been attempting to convert autologous anti-BCMA CAR-T therapy into allogeneic CAR-based immune cell therapy. As shown in Figure 1, allogeneic CAR-based immune cell therapy is independent of patients’ own cells. By starting with the collection of healthy donor blood, specific immune cells are then isolated, genetically modified, and expanded in vitro. Ideally, these cells undergo necessary quality control (QC) testing and are stored for future use. One of the key advantages of allogeneic cell therapy is its potential to be applied “off-the-shelf”. In this scenario, patients are not required to wait for the personalized cells to be manufactured and need not be concerned about whether their cells will qualify for the treatment. Additionally, by creating an inventory of healthy donor-derived CAR-based immune cells, the cost per patient can be significantly reduced [36]. However, achieving this goal is not easy. The widely applied T cells for CAR-based immune cell manufacturing nowadays are known as αβ T cells, which refer to T cells bearing the TCR α/β that represent the majority (~95%) of the peripheral blood-circulating T cell population [37]. Unfortunately, this type of cell source is not suitable for allogenic use due to potential alloreactivity caused by the recognition by TCR of peptide-allogeneic MHC complexes [38]. In this review, taking anti-BCMA CAR-based immune cell therapy as an example, we discuss the immune cells that are currently being explored for allogeneic application in treating MM, such as gene edited-αβ T cells, γδ T cells, and NK cells. We hope that the immune cells we mention here could also provide insights for those who wish to develop allogeneic cell therapies for other types of cancers or target other cell antigens.

## 2. Gene-Edited-αβ T Cells

**Figure 2:**
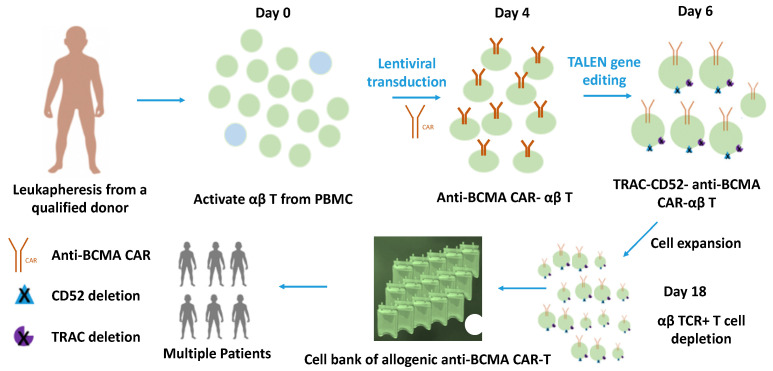
The general cell manufacturing process of gene edited-αβ T cells.

Inactivation of the endogenous TCR by genome engineering methods is one of the most promising strategies for generating αβ T cells for allogeneic use. For example, ALLO-715 is a type of genetically modified anti-BCMA CAR-T cell that uses TALEN technology to disrupt the T-cell receptor alpha constant gene (TRAC) to reduce the risk of graft-versus-host disease (GvHD) (Figure 2) [39]. Additionally, the CD52 gene of ALLO-715 is knocked-out to allow for the use of an anti-CD52 monoclonal antibody (mAb) for selective and transient lymphodepletion (LD) for the patient prior to CAR-T treatment [39]. ALLO-715 is currently being evaluated in a Phase I clinical trial (NCT04093596) for adult patients with r/r multiple myeloma who have failed with three prior lines of therapy; patients receive ALLO-715 at one of four dose levels (40, 160, 320, and 480 × 10^6^ CAR+ T cells) post-LD [39]. As of 14 October 2021, the interim report showed that in 43 treated subjects, no GVHD events had been observed, 53% had developed Grade 1 and 2 Cytokine Release Syndrome (CRS), and 14% had experienced low-grade neurotoxicity [40]. The overall response rate among the 320 × 10^6^ dose cohort was 71% [40]. ALLO-605 is another allogeneic anti-BCMA CAR-T cell from the same company, Allogene Therapeutics, and was granted by the FDA with Fast Track designation in June 2021 and orphan-drug designation in April 2022 [41]. Compared to ALLO-715, ALLO-605 is equipped with an additional Constitutively Active Chimeric Cytokine Receptor (CACCR) to further regulate T cell exhaustion and improve T cell function and potency [42]. Currently, this product is also under a Phase 1 clinical trial evaluation with similar patient inclusion criteria as that for ALLO-715 [41,43]. The other gene edited-αβ T cell products targeting BCMA for MM treatment, CTX120, are produced using the CRISPR/Cas9 system to eliminate TCR and MHC class I, coupled with specific insertion of the CAR at the TRAC locus [44]. These genetically modified T cells demonstrated desired in vivo persistence and anti-tumour effects in MM mouse models, which is currently being investigated in a Phase I clinical trial for r/r MM patients who have been treated with at least two prior lines of therapy [44,45].

Nevertheless, engineered endonucleases for genetic editing such as the CRISPR/Cas9 system may bring potential off-target risks. Researchers have attempted to optimize the nuclease design [46]. Recently, Madison et al. reported the application of the high-fidelity RNA-guided endonuclease Cas-CLOVER for T cell receptor beta constant (TRBC) and β2 microglobulin *(B2M)* gene editing and the *piggyBac* transposon for CAR delivery. They successfully produced allogeneic anti-BCMA CAR-T cells composed of high percentages of stem cell memory T cells (over 60% with some donors) and exhibited potent anti-tumour cytotoxicity against MM.1S cells in xenograft mouse MM models [47]. Specifically, Cas-CLOVER consists of a fusion of catalytically dead SpCas9 with the nuclease domain from a Clostridium Clo051 type IIs restriction endonuclease. Cas-CLOVER is activated upon the dimerization of the Clo051 nuclease domain by RNA-guided recognition of two adjacent 20-nucleotide target sequences. Compared to the paired nickase approach with a Cas9-D10A mutant, Cas-CLOVER does not induce double-strand break (DSB) or nick and has lower off-target nuclease activity with off-target indel rates among donors ranging between 0.012% and 0.089% [47]. However, further head-to-head fidelity comparisons between various gene editing tools and extensive safety evaluations for “off-the-shelf” clinical applications must be performed. Another strategy to avoid the potential risk of genomic modification is to knock down TCR expression at the mRNA level. Ceylad Oncology has generated such allogeneic anti-BCMA CAR-T cell products (CYAD-211) by applying short hairpin RNA [48]. In the ongoing Phase I exploratory trial IMMUNICY-1 for treating r/r MM patients with at least two prior MM treatment regimens that include exposure to IMiD and PIs either alone or in combination, CYAD-211 has achieved 2/8 partial responses and 5/8 stable diseases in low dosage levels with no GvHD observed, suggesting the potential of applying this technology in allogeneic settings [48].

## 3. γδ T Cells

γδ T cells represent a minor subset of the total CD3+ T cell population (1–5%) and express a pair of Vγ chain (Vγ 2, 3, 4, 5, 8, 9, or 11) with Vδ (Vδ1, 2, 3, or 5) chain [49]. Unlike its counterpart αβ T cells, γδ T cells could recognize antigens in a human leukocyte antigen (HLA)-independent fashion [42]. For instance, Vγ9Vδ2 T cell receptors can recognize the endogenous isopentenyl pyrophosphate (IPP), which is typically overexpressed in cancer cells due to the dysregulated mevalonate pathway, with the help of butyrophilin (BTN) 3A1 and BTN 2A1 [50]. Currently, most γδ T cell-based immunotherapies focus on two major subtypes: Vδ1 and Vδ2. The Vδ1 T cell subset resides in tissues while Vδ2 T-cells are mostly present in the circulation and can be expanded in vitro from peripheral blood sources [42]. Multiple groups have tested the therapeutic potential of unmodified Vγ9Vδ2 T cells either by systemic activation using aminobisphosphonates/phosphoantigens or adoptive transfer of in vitro expanded cells [51,52,53,54,55,56]. Both methods demonstrated outstanding safety profiles of Vγ9Vδ2 T cells in both autologous and allogeneic settings but were mostly associated with limited clinical benefits in a number of lymphoid malignancies and solid tumours such as renal cell carcinoma, lung cancer, and pancreatic cancer [51,52,53,54,55,56]. For example, the administration of zoledronate-activated Vγ9Vδ2 T lymphocyte-activated killer (LAK) cells to patients with multiple myeloma who had been previously treated with chemotherapy was tested and M protein levels remained at baseline in four out of six patients [55]. However, other subtypes of γδ T cells might perform differently. IN8Bio Inc. reported three cases from the Phase I clinical trial of INB-100, which is a matched donor-derived γδ T cell (the Vγ and Vδ chains were not specified) therapeutic candidate in development for patients with leukemia undergoing a haploidentical hematopoietic stem cell transplant (HSCT). Two of the INB-100 treated patients had been in remission for over two years and the third patient was in continuing remission for over a year post-treatment with no emergent serious adverse events [57].

More recently, some groups began to investigate genetically modified γδ T cells, especially the anti-tumour potential of CAR-expressing γδ T cells. CAR-γδ T cells are currently being studied in clinical trials, but none of them are designed to target BCMA yet. Adicet Bio is evaluating ADI-001, the first-in-class allogeneic γδ CAR T-cell therapy targeting the B-cell antigen CD20 in adult patients with relapsed/refractory advanced B-cell lymphoma [58]. As of 14 February 2022, among six evaluable patients, no GvHD was reported and the CR rate was 67% (4/6) [58]. CytoMed Therapeutics has also started two Phase I clinical trials using allogeneic CAR-γδ T cells targeting NKG2DL in treating various solid cancers (NCT04107142 and NCT05302037), although no result has been published yet. In terms of pre-clinical testing of anti-BCMA CAR-γδ T cells, some promising pre-clinical results have been reported very recently. In 2022, Zhang X et al. demonstrated the generation of in vitro expanded Vγ9Vδ2 T cells expressing anti-BCMA CAR via mRNA electroporation [59]. The modified Vγ9Vδ2 T cells displayed a highly specific killing activity against BCMA-expressing MM cell lines in vitro and in KMS-11 xenograft mouse models [59]. The treatment of the tumour-bearing mice with zoledronic acid (ZOL) and anti-BCMA CAR-Vγ9Vδ2 T cells resulted in a significant reduction of tumour burden and prolonged survival [59]. It would be worthwhile to further test Vδ1 T cells or stem cell-derived γδ T cells as backbones for anti-BCMA CAR development [60,61].

Due to the low abundance and innate regulatory functions of γδ T cells, how to improve in vitro expansion capacity, gene editing efficiency, and effector functions has become a major issue for further clinical applications of γδ T. Apart from the classical approach using zoledronic acid and IL-2 for Vδ2 T cell expansion, multiple other strategies have been tested. For example, a two-step expansion protocol for Vδ1 T cells was previously described with selected cytokines including IL-15 [62]. Other expansion protocols such as utilizing mitogen phytohemagglutinin (PHA) plus IL-7 stimulation for highly cytolytic Vδ1 expansion have also been developed [63]. Polyclonal γδ T cells have been successfully established using 4-1BBL-expressing artificial antigen-presenting cells (APCs) and IL-2 plus IL-21 [64]. However, in this case, the application of tumour cell-originated aAPCs for rapid cell expansion might pose additional regulatory filing difficulties. Another difficulty is how to enhance the gene editing efficiency of γδ T cells. The literature has shown that gene editing efficiency is comparably low for γδ T cells using the lentiviral transduction method [65]. Additional research must be conducted for the optimization of γδ T cell transduction protocols and other non-viral strategies must be explored. For example, the mRNA transfection for transient CAR expression and DNA transposon systems (e.g., *piggyBac*, *Sleeping Beauty*, and *Tol2*) for stable CAR expression could be important avenues to consider. Moreover, improving the persistence of infused γδ T cells in patients post-infusion is critical to sustaining their effector functions. For example, direct infusions of ZOL in combination with low-dose IL-2 are currently widely applied to activate and expand tumour-reactive Vγ9Vδ2 cells in vivo [66]. However, some studies have shown that repeated ZOL application could result in a progressive decline of Vγ9Vδ2 T cells in vivo [67]. Other strategies such as ectopic expression of cytokines by γδT cells themselves could be considered.

## 4. NK Cells

NK cells, as a type of innate lymphoid cell that is primarily involved in early pathogen infection control and anti-tumour immunity, have also been explored and developed as another cell source for targeting MM with anti-BCMA CAR constructs [68,69,70].

NK cells can be obtained from various origins. One example of these cells is primary NK cells, which typically take up 5–20% of peripheral blood mononuclear cells (PBMCs) and up to approximately 30% of cord blood (CB) lymphocytes in the human body [71,72]. Recently, NK cells have also been shown to be capable of differentiating from both embryonic stem cells (ESCs) and induced Pluripotent Stem cells (iPSCs) in vitro, which could potentially solve problems regarding heterogeneous cell population and insufficient donors for primary NK cells [73,74]. NK cancerous cell lines (e.g., NK-92) are the other option, although gamma irradiation is typically required prior to clinical infusion of these cells to prevent any undesired in vivo expansion and tumourigenesis [75].

Unlike αβ T cells, since NK cells’ anti-cancer mechanisms do not rely on HLA matching and antigen exposure and recognition, NK cells are known to be “naturally cytotoxic” and could be applied allogenically [76]. The adoptive transfer of allogeneic NK cells in treating malignancies has already been shown to be safe in several clinical trials with very limited GvHD and other side effects observed, including leukemia and solid cancers [77,78].

NK cells equipped with CARs have already been examined in targeting various antigens for many cancer types with encouraging results. For example, in 2019, non-viral transfected CAR-NK cells that target NKG2D ligands were used for treating three metastatic colorectal cancer patients in a pilot clinical trial study. No severe side effects were observed in all patients and the number of tumour cells in ascites was almost undetectable at the end of treatment for two of these patients [79]. The other study administered retroviral-transduced anti-CD19 CAR-NK cells to 11 patients with r/r CD19-positive cancers (non-Hodgkin’s lymphoma or chronic lymphocytic leukemia [CLL]). Seven of them achieved CR and no CRS, neurotoxicity, or GvHD was detected in any patients [80].

In terms of treating MM, anti-BCMA CAR-NK cells seem to be less prevalent; this is likely due to the pre-existing autologous anti-BCMA CAR-T cells in the market [16]. Currently, there are only three clinical trials that can be found on the clinicaltrials.gov website (NCT05008536, NCT03940833, NCT05182073) and none of them have published any data yet. Interestingly, these trials chose three different origins of NK cells: CB, NK-92, and iPSCs, which may represent distinct clinical considerations while they were designing the study. A few pre-clinical studies, however, have demonstrated the competency and potential of anti-BCMA CAR-NK cells for treating MM. As reported by Ng et al., the first-generation mRNA-transfected anti-BCMA CAR-NK cells showed superior in vitro cytotoxicity and IFN-γ releasing and degranulation capacities compared to control NK cells upon the activation of MM cell lines [81]. Furthermore, a significant tumour burden reduction was also observed after injecting these cells intravenously into an MM xenograft mouse model [81]. Another study conducted by Martin et al. transduced an NK-92 cell line by lentivirus with a second-generation anti-BCMA CAR. The anti-BCMA NK-92 cells showed superior cytotoxicity against U2266, XG-1, and K562 cell lines compared to the parental NK-92 cell line and no toxicity against PBMCs from healthy donors was observed for these CAR-equipped NK-92 cells [82].

To further facilitate anti-BCMA CAR-NK cell clinical applications, three major challenges must be carefully addressed. One hurdle is the genetic modification for primary NK cells to stably express CARs. Although it might be comparably manageable for cancerous cell lines and iPSCs, it is often difficult to transduce primary NK cells using viral vectors that are widely applied for CAR-T cells, such as lentivirus or retrovirus, due to their intrinsic anti-viral self-defense mechanisms [83,84,85]. Therefore, non-viral transfection systems could be a better option in this case for NK cells’ genetic modification. Recently, a group of researchers successfully transfected primary NK cells by the *piggyBac* transposon system with an NKG2D CAR construct; the cells were shown to achieve long-term stable transgene expression even after 49 days, with approximately 90% CAR-expressing NK cells [86]. More importantly, these CAR-NK cells also exhibited potent in vitro and in vivo anti-cancer effects, revealing their potential for application in clinical settings [86]. The second concern is the possible in vivo infiltration problem of CAR-NK cells into cancer target sites after intravenous injection [87,88]. As reported by many studies, in vitro manipulation could lead to down-regulation of the homing receptors on NK cells that are essential for their trafficking [89,90,91]. One applicable strategy to solve this problem is to enhance chemokine receptors’ expression in NK cells. As demonstrated by Ng et al., the transgenic expression of CXCR4 (a chemokine receptor) significantly improved the migration and bone marrow homing capacities of NK cells [81]. Additionally, the co-expression of transgenic CXCR4 and anti-BCMA CAR in NK cells was shown to further strengthen their cancer-eradicating effects in a MM xenograft mice model compared to CAR-NK cells without CXCR4 transfection [81]. The short lifespan and persistence of CAR-NK cells in vivo is the last obstacle [92]. Without cytokines’ support, CAR-NK cells became barely detectable in the peripheral blood after 7 days [93]. A few approaches were developed to address this issue, including direct in vivo exogenous cytokine injection (e.g., IL-2), ectopic cytokine co-expression (e.g., IL-15), and pre-induction of cytokine-induced memory-like (CIML) NK cells with IL-12, IL-15, and IL-18 [94,95]. One exciting example which has already reached the clinical trial stage utilized a retroviral construct co-expressing anti-CD19 CAR and IL-15 to generate CAR-NK cells [80,96]. These anti-CD19 CAR-NK cells were capable of expressing IL-15 ectopically and showed enriched proliferation, persistence, homing, and cytotoxicity against tumour targets in vivo compared to those without IL-15 expression [96].

## 5. Other Potential Candidates (e.g., Macrophages, iNKT Cells, etc.)

Other than the cell sources mentioned above, although not very predominant, there are a few other types of immune cells that are beginning to draw the attention of the scientific community for developing CAR immunotherapy, such as invariant Natural Killer T (iNKT) cells, macrophages, dendritic cells, B cells, etc. [97]. A few of these have entered clinical trials with various CAR constructs for different types of cancer, although most of them are still under pre-clinical development [97]. For anti-BCMA CAR application in MM, to our knowledge, minimal investigations have been conducted thus far. One study equipped iNKT cells with anti-BCMA CAR through repeated retrovirus transduction and achieved a mean transduction efficacy of approximately 50% [98]. These genetically modified iNKT cells demonstrated CAR-dependent cytotoxicity against the MM cell line UM9 and a low production level of IL-6 in vitro, which is one of the unfavored cytokines involved in CRS [98]. iNKT cells are a special subset of T cells. Similar to Vγ9Vδ2 T cells, they could be used allogenically because they do not interact with the peptides presented by MHC molecules for their cytolytic functions but with the glycolipids presented by a non-polymorphic MHC-I-like molecule, named CD1d [99]. iNKT cells only account for 0.003–0.7% of CD45+ cells in peripheral blood [100]. Although α-galactosylceramide (α-GalCer) was found to be effective in stimulating iNKT cells in in vitro environments, the comparably sophisticated isolation procedure, cell purity control, and clinically relevant scale expansion of iNKT cells still remain the key challenges hindering the application of iNKT cells in MM treatment [98]. Given the above-listed limitations, in our opinion, iNKT cells may not be able to outcompete the other allogeneic immune cell candidates as the cell source for CAR implementations at the current stage. For the other potential candidates such as macrophages, DCs, etc., much more research must be conducted to determine their suitability for MM treatment.

## 6. Current Landscape of Clinical Trial Regulations: Autologous vs. Allogeneic CAR Immune Cells

Currently, no regulations or guidelines have been specifically enacted for different types of CAR immune cells entering clinical trials. Instead, all CAR-equipped immune cells are generally categorized as genetically modified cell products and regulated similarly to the autologous CAR-T cell products on the market. Compared to therapeutic chemistry entities and other biologicals, the regulations for these cell products possess the following characteristics: Cell products have more stringent requirements on raw material source control and manufacturing in process control. For example, all cell lines, including feeder cells used in manufacturing, should have clear source and cell passage history with controllable risks [101]. If irradiated cells are involved in the product, the information of the irradiator should be documented and there must be data showing that the irradiated cells maintain their expected characteristics [102]. However, the follow-up time for cell therapy-treated patients is comparably longer, which is typically recommended to be up to 15 years after the treatment, possibly due to the unknown medical risks involved [103,104]. If the genetically modified CAR immune cells are intended to be used allogenically, additional regulations are typically required.

In the US, allogeneic CAR immune cells are generally regulated as gene therapy and human cell products. The US FDA released Chemistry, Manufacturing, and Control (CMC) Information for Human Gene Therapy Investigational New Drug Applications (INDs) in 2020. Per this guidance, cells for allogeneic use should be tested as required by 21 CFR Part 1271 and screened for risks such as HIV, HBV, HCV, human TSE, and syphilis. If cells are from a single donor for better consistency, it is recommended that qualified master and working cell banks be established. The sample should be sufficient for tests of qualification and adventitious agents, and related information should be provided in IND application [102]. In March 2022, the US FDA published a new draft guideline specifically for genetically modified lymphocyte products (e.g., CAR-T, CAR-NK, and CAR-B): Considerations for the Development of Chimeric Antigen Receptor (CAR) T Cell Products Draft Guidance for Industry. In this guidance, GvHD was specially emphasized for allogeneic products; its information should be collected in early-phase studies and considered regarding Dose Limiting Toxicities (DLT) and stopping rules. Moreover, a protocol is recommended to describe the immunological matching of the donor and the recipient [103].

In Europe, the European Medicines Agency (EMA) regulates allogenic cell-based medicinal products with Guideline on Human Cell-Based Medicinal Products and Guideline on Quality, Non-Clinical and Clinical Aspects of Medicinal Products Containing Genetically Modified Cells. In these guidelines, both master and working cell banks should be well characterized and established if cell lines are used. For allogeneic cell origins, if possible, histocompatibility markers and genetic polymorphisms should be identified [105]. Moreover, an in vitro insertion site analysis should be performed before releasing allogeneic products [104].

The China National Medical Products Administration (NMPA) also published Guidance for Pharmacological Research and Evaluation on Immune Cell Therapy in May 2022. With regards to allogeneic cell products, this guidance emphasizes that the source of donor cells should follow laws, regulations, and ethical guidance. Donors’ information should be collected, including age, gender, known medicinal history and exposure to radiation, past medicinal history, family histories, microorganism and pathogen screening information, HLA typing, blood type, complete blood count, etc. [101].

In summary, although various regions or countries may adopt slightly different regulatory requirements for allogeneic CAR immune cell products, the general concept is to keep these products safe for patients. However, since all these products are new to the market, the relevant regulations and guidelines are also in their preliminary stages. More detailed and completed versions are expected to be released in the near future.

## 7. Conclusions and Perspectives

**Figure 3:**
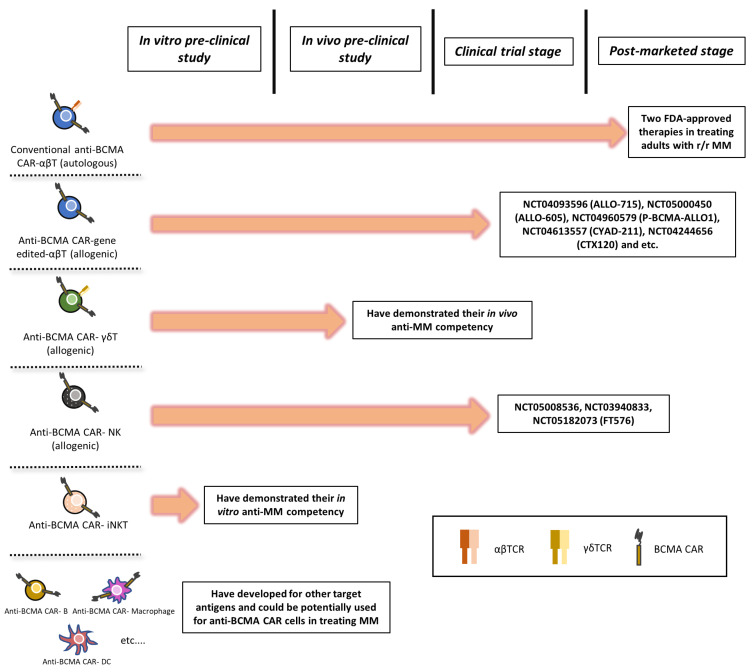
The current development stages of major types of anti-BCMA CAR-based immune cells.

To conclude, Figure 3 summarizes the development stages of major immune cell sources that are currently used for anti-BCMA CAR therapy in treating MM. As shown, although the autologous CAR-T cells remain the only approved therapy on the market, there are a number of other allogenic immune cell sources that are catching up forthwith.

To further push these CAR-based therapies forward to benefit more patients, when and how these CAR-based immune cells will be administrated are also worthy of investigation. As mentioned at the beginning, the current two FDA-approved anti-BCMA autologous CAR-T cell products, ABECMA and CARVYKTI, are only permitted to be used as a single agent for patients with r/r MM after at least four lines of therapy, including a proteasome inhibitor, an immunomodulatory agent, and an anti-CD38 monoclonal antibody [28,29]. Similarly, most of the allogenic gene edited-αβ T cells in the ongoing clinical trials were also used for r/r MM patients as a single treatment. One possible strategy to improve this treatment is to combine CAR-based immune cell therapy with other standard therapies for earlier-stage patients. Indeed, there is an ongoing clinical study can be found online (NCT04287660), which combines BiRd regimen (clarithromycin, lenalidomide, dexamethasone) with autologous anti-BCMA CAR T cells for treating newly diagnosed MM patients. However, there is still a long process to bring CAR-based immune cell therapy to the front line for MM treatment or even for the treatment of other types of cancers (instead of a “last-resort” treatment). This is not only due to regulatory difficulties but also the complicated interactions and interferences between different drugs/treatments, which must be carefully studied to better understand optimal dosages, toxicity, and side effects.

Given the above-mentioned advantages and the current development progression, in our opinion, the future of allogenic anti-BCMA CAR immune cell therapy is favored and worthy of continued investment. However, the focus, for now, might not be to completely replace the existing treatment. Instead, how to integrate the cell therapy into the current treatment algorithms and make it more cost-effective could be the avenue to consider.
